# COVID-19-Induced Thrombocytopenia: A Brief Literature Review and Case Report

**DOI:** 10.7759/cureus.30993

**Published:** 2022-11-01

**Authors:** Essam K Nagori, Henrik Ghantarchyan, Aftab Qadir, Sarkis Arabian

**Affiliations:** 1 Internal Medicine, Arrowhead Regional Medical Center, Colton, USA; 2 Critical Care, Arrowhead Regional Medical Center, Colton, USA

**Keywords:** adamts13, platelet, itp, covid-19, thrombocytopenia

## Abstract

With sporadic surges of COVID-19, medical professionals are continuously expanding their knowledge and contributing to medical literature through experiences and research. We present a rare case of a 65-year-old Hispanic male diagnosed with COVID-19-induced immune thrombocytopenic purpura (ITP). Commonly seen in cases with COVID-19-vaccine-induced thrombocytopenia, there are very few published case reports of ITP as a result of the COVID-19 virus.

## Introduction

Thrombocytopenia is commonly encountered in acutely ill hospitalized patients and is known to originate from various etiologies [1]. The complications associated with SARS-CoV-2 seem to be ever-growing. The risk of thromboembolic disease is already well established with this virus, and the current understanding is that COVID-19 leads to a hypercoagulable state [2]. It is known that a mild thrombocytopenia state can exist in approximately 70%-95% of COVID-19 patients [3]. Thrombocytopenia, however, is well documented in the original SARS and the Middle East respiratory syndrome (MERS) as well, with platelet levels indicative of disease severity [4].

There have been a few publications on the development of severe thrombocytopenia and more specifically the development of immune thrombocytopenia purpura (ITP) secondary to SARS-CoV-2. Current literature attempts to shed some light on this rare phenomenon [5,6]. ITP is a state of platelet destruction caused by antibodies directed against antigens on platelets and megakaryocytes, likely from an autoimmune etiology [1]. ITP is known as a diagnosis of exclusion and is a rare complication of COVID-19 infection [7]. We present a case of a 65-year-old Hispanic male who presented to our institution with epistaxis, oral mucosal bleeding, melena, cough, and fever and who was ultimately diagnosed with COVID-19-induced ITP.

## Case presentation

A 65-year-old male with a history of hyperlipidemia, hypertension, insulin-dependent type 2 diabetes mellitus, stage 5 chronic kidney disease (CKD) on hemodialysis (HD), and colon cancer in remission presented to the emergency department (ED) for one week of worsening cough and fever. On presentation, he was found to be positive for COVID-19. Additionally, he complained of bleeding oral ulcers, epistaxis, and melena for two days. This was accompanied by right-sided facial pain and swelling. The worsening bleeding was what prompted him to visit the ED. Of note, the patient was vaccinated for COVID-19, having completed the two-dose series of Pfizer-BioNTech (Pfizer, New York, NY, USA), approximately one month earlier.

Two years earlier, he was diagnosed with colon cancer and subsequently treated with resection. A repeat colonoscopy was done a year earlier without any signs of recurrence. He had known stage 5 CKD with preserved urine production and was not on regular HD although he had an arteriovenous (AV) fistula on the left upper extremity.

On initial presentation, his vital signs were stable. On physical examination, there were no obvious signs of overt bleeding; however, minor petechiae were noted on his lower extremities. He was found to have a leukocytosis of 15 × 10^3 ^µL^-1^ with no bands. Additionally, his hemoglobin (Hgb) was noted to be 7 g/dL, decreased from 9 g/dL, and a platelet count of 4 × 10^3 ^µL^-1^, decreased from 255 × 10^3^ µL^-1^ one month earlier. His immature platelet fraction was 0.0. The prothrombin time (PT) and activated partial thromboplastin time (aPTT) are shown in Table 1. Troponin was significant at 0.67 ng/mL with electrocardiogram findings of T-wave inversions in V2 and V3. He was also found to be positive for SARS-CoV-2. A maxillofacial CT with contrast was obtained as the patient was complaining of facial pain and swelling, which was negative for abnormalities or acute pathology. He was then admitted to the internal medicine service for the management of type 2 myocardial infarction, gastrointestinal bleeding (GIB), thrombocytopenia, and COVID-19 pneumonia. As sepsis ensued, the patient was started on broad-spectrum antibiotics due to tachycardia and hypotension. Blood cultures obtained were negative for growth. Due to the recommendations at the time, we treated the patient with baricitinib, dexamethasone, and remdesivir in addition to supplemental oxygen.

**Table 1:** Significant laboratory results on presentation.

Blood test results (units)	Patient value	Reference range
White blood cells (10^3^ µL^–^^1^)	15	4.5-11.1
Hemoglobin (g/dL)	7	13.0-17.0
Platelets (10^3^ mL^–^^1^)	4	120-360
Immature platelet fraction (%)	0.0	0.9-11.2
Prothrombin time (s)	17.2	11.8-14.2
Activated partial thromboplastin time (s)	49.9	25.4-36.8

The patient was started on HD via his AV fistula. The next day his platelet count decreased to 2 × 10^3^ µL^-1^, and he was transfused one unit of platelets. He continued to exhibit epistaxis and oral mucosal bleeding. The patient was seen by hematology/oncology and was started on high-dose steroids for ITP. Once steroids were started, the patient developed persistent hyperglycemia, likely secondary to high-steroid dosage, and was thus upgraded to the ICU for a continuous insulin infusion. On day 3 of hospitalization, the patient had an acute change in mental status and eventually was noted to have apneic episodes, necessitating emergent intubation. Laboratories were significant for an Hgb of 4.2 and platelets of 6 at that time. A thromboelastogram (TEG) showed diminished maximal amplitude (MA) but was otherwise normal (Table 2). A stroke protocol was initiated and a noncontrast CT scan of the head was negative for any bleeding or ischemia.

**Table 2:** Significant laboratory results of TEG. K-time, coagulation time; R-time, reaction time; TEG, thromboelastogram

Blood test results	Patient value	Reference range
Kaolin R-time (min)	7.2	4.6-9.1
Kaolin K-time (min)	0.9	0.8-2.1
Alpha angle (degrees)	77.2	63-78
Rapid maximal amplitude (mm)	40.1	45-69

**Figure 1:**
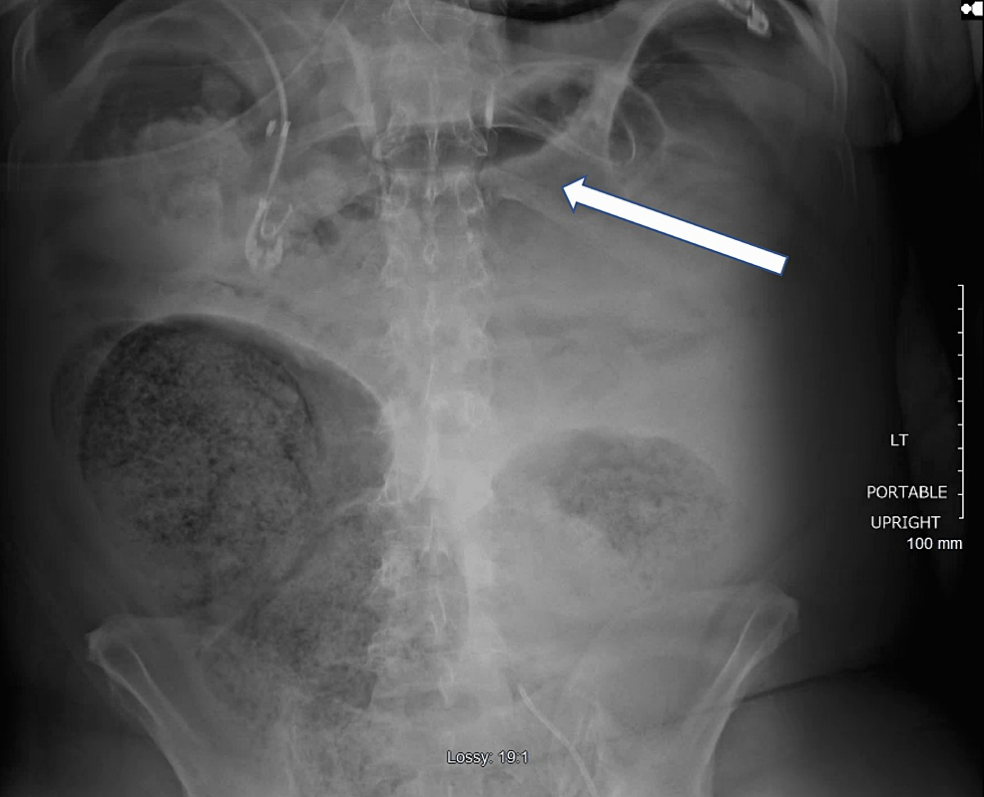
Plain upright abdominal plain film radiograph indicating free air under the diaphragm (white arrow).

Once on mechanical ventilation, the patient required vasopressor support; therefore, he was switched from intermittent HD to continuous renal replacement therapy. At this time, the patient received three units of plasma, three units of packed red blood cells, and one unit of fresh frozen plasma (FFP). Over the next few days, the patient improved and was extubated on day 8. On day 11 of hospitalization, the patient had an aspiration event and worsening mentation, requiring reintubation. On the same day, while receiving HD, the patient had a bradycardic arrest and required advanced cardiac life support (ACLS). Return of spontaneous circulation was achieved after successful ACLS. Subsequently, the patient required the titration of three vasopressors, which included Levophed, vasopressin, and epinephrine. Four days after ACLS measures were taken, an upright abdominal plain film radiograph, taken for abdominal distention, indicated free air in the abdomen (Figure 1). General surgery was consulted, and the decision to take the patient to the operating room was made after a discussion with the family of risks, given his underlying comorbidities and current critical state. At this time, his platelets had improved to 41 × 10^3^ µL^-1^ after one unit of FFP transfusion. In the operating room, he was found to have multiple perforations throughout the colon with >1 L of feculent material and necrotic bowel throughout. Surgeries included subtotal colectomy with small bowel resection at the terminal ileum, which was left in discontinuity, and the patient was brought back to the ICU with an open abdomen in guarded condition. During the entire hospitalization, our patient required a total of 11 units of platelets, six units of plasma, and nine units of packed red blood cells. Given the poor prognosis, his family elected to change to comfort care, and he expired shortly thereafter.

An ADAMTS13 test was sent during hospitalization; however, results returned after the patient's death. ADAMTS13 was noted to be at 51%, aiding the confirmation of our diagnosis of ITP.

## Discussion

ITP is a known but rare complication to arise from COVID-19 infection, with several published case reports and case series [8,9]. Currently, the mechanism of development is unclear but has been postulated to include cytokine storm; direct marrow infection, and hence, decreased platelet production; autoimmune dysregulation; and direct platelet aggregation and consumption [10]. Our patient had a platelet nadir of 2,000 μL^-1^ and a peak of 118,000 μL^-1^, requiring a total of 11 units of platelets, six units of plasma, and nine units of packed red blood cells during the hospitalization period.

When considering differential diagnoses, it is important to consider vaccine-induced thrombotic thrombocytopenia (VITT) and thrombotic thrombocytopenic purpura (TTP). After the emergence of lifesaving COVID-19 vaccines, there has been an increasing number of VITT cases reported. It was found that it occurs 5-30 days after the first dose. Its popularity has been progressively increasing since its first announcement on social media on March 19, 2021 [11]. There were 367 cases documented after the first vaccine and 44 cases after the second AstraZeneca-Oxford vaccine (AstraZeneca, Cambridge, UK). This was also observed in 12 cases after the Johnson & Johnson vaccine. VITT is a prothrombotic case in which patients are found to have episodes of a stroke, pulmonary embolism, deep-vein thrombosis, or splanchnic vein thrombosis, none of which was observed in our patient [11]. Additionally, VITT is a likely diagnosis if a patient is found to be thrombocytopenic, has a D-dimer > 4,000 FEU and is more than eight times the upper limit of normal, and symptoms are not better explained by COVID-19 [12]. This was not observed in our case. Although the patient was recently vaccinated, he was found to be acutely infected with COVID-19, had a D-dimer of 1,940 FEU.

Another coagulopathy that was considered in our differential diagnoses included TTP. Alhomoud et al. reported a case of COVID-19-induced TTP, with an additional six cases found after a PubMed and Google Scholar search, resulting in a total of seven known cases of TTP followed by COVID-19 [13]. TTP can be either acquired or hereditary, resulting from either antibodies to ADAMTS13 or a congenital deficiency of ADAMTS13. Patients with TTP are found to have an ADAMTS13 level <10% [13]. This can commonly be caused by viral infections, causing a prothrombotic state, manifesting as microvascular thrombosis [11,13]. Our patient was found to have an ADAMTS13 level of 51%, excluding the diagnosis of TTP, and was not found to have any prothrombic manifestations.

## Conclusions

This case highlights the necessity to be vigilant in patients with COVID-19 infection as this disease can present not only with thromboembolic complications more commonly but also with progressive thrombocytopenia due to ITP. Early recognition of the lesser-known complications of the disease will help keep clinicians from missing potentially fatal complications.
